# A Narrative Review of Kenya’s Surgical Capacity Using the Lancet Commission on Global Surgery’s Indicator Framework

**DOI:** 10.9745/GHSP-D-21-00500

**Published:** 2022-02-28

**Authors:** Hugh Shirley, Richard Wamai

**Affiliations:** a Harvard Medical School, Boston, MA, USA.; b Department of Cultures, Societies and Global Studies, Northeastern University, College of Social Sciences and Humanities, Integrated Initiative for Global Health, Boston, MA, USA.

## Abstract

Surgery, anesthesia, and obstetric (SAO) care is quickly being recognized for its critical role in cost-effectively improving global morbidity and mortality. Six core indicators for SAO capacity were established in 2015 by the Lancet Commission on Global Surgery (LCoGS) and include: SAO provider density, population proximity to surgery-ready facilities, annual national operative volume, a system to track perioperative mortality rate, and protection from impoverishing and catastrophic expenditures. The surgical capacity of Kenya, a lower-middle-income country, has not been evaluated using this framework. Our goal was to review published literature on surgery in Kenya to assess the country’s surgical capacity and system strength. A narrative review of the relevant literature provided estimates for each LCoGS indicator. While progress has been made in expanding access to care across the country, key steps remain in the effort to provide equitable, affordable, and timely care to Kenya’s population through universal health coverage. Additional investment into training SAO providers, operative infrastructure, and accessibility are recommended through a national surgery, obstetric, and anesthesia plan.

## Abstract (precis)

Limited progress has been made on the expansion of access to surgical care in Kenya as assessed with the Lancet Commission on Global Surgery’s indicator framework, underscoring the need for a national surgery, obstetrics, and anesthesia plan.

## Teaser

**Key Findings:**

- While more than 90% of Kenya’s population lives within 2 hours of a health care facility, this statistic neglects reasons other than distance, such as socioeconomic factors, that prevent a patient from reaching timely surgical care, especially if nearby hospitals are not equipped for certain surgeries.
- Out-of-pocket expenses for perioperative needs, including transportation, food, and housing, as well as lost productivity for the patient and any caretakers, contributes to a persistently high risk of impoverishing or catastrophic expenditure for surgical patients.

**Key Implications:**

- A standardized, international framework with metrics that indicate success is needed for the evaluation of progress in surgery, obstetrics, and anesthesia infrastructure, but the incorporation of national priorities and measures of success is critical to achieve equitable development.

## INTRODUCTION

Safe, timely, and affordable surgery is an essential right for human health. An estimated 17.5 million additional surgical procedures are required annually to meet surgical need in Eastern sub-Saharan Africa; however, the true number is likely greater given inherent underestimates when extrapolations across countries are made.^1^ In Kenya, a lower-middle-income country of 52.5 million people, access to surgery is an important lever for elevating the standard of care across the country.^2^^,^^3^ Here, we examine Kenya’s standing and progress toward the goals laid out within 6 key indicators as well as those set by national policies and discuss additional challenges and barriers to achieving equitable and sustainable surgery throughout the nation’s devolved health care system.

The 2015 Lancet Commission on Global Surgery (LCoGS) outlined 6 indicators with targets that signal strong national surgical programs.^3^ The indicators and their international 2030 targets (Table) outline necessary surgical infrastructure, personnel, and outcomes and have been suggested as part of a basic reporting package for surgery by the World Health Organization.^4^ The indicators are divided across 3 subgroups: preoperative, perioperative, and postoperative. The preoperative targets are twofold. First, 80% of the population is within 2 hours of a hospital that is equipped to perform the 3 bellwether procedures: laparotomy, cesarean delivery, and open fracture care. Second, achievement of 20 surgery, anesthesia, and obstetrics (SAO) personnel per 100,000 population. Perioperative targets are 5,000 surgical procedures per 100,000 population annually and the establishment of a national system for tracking the perioperative mortality rate (POMR) and surgical volume. Post-operative targets include 100% protection against impoverishing expenditures (IE) and catastrophic health care expenditures (CHE) resulting from surgery.

**TABLE.** Estimates for Each Lancet Commission on Global Surgery Indicator in Kenya^a^

	**Indicator**	**Goal**	**Estimates**
Preoperative	Surgical workforce density	20 per 100,000 population	2.35^21^
	Access to essential surgical care	80% of population within 2 hours of a facility capable of performing 3 bellwether procedures	>90%^16^
Perioperative	POMR system	Presence of a national POMR system	Absent
	Surgical volume	5,000 procedures per 100,000 population	252^26^–11,110^27^
Postoperative	Risk of impoverishing expenditure	100% protection	63.6%^36^ 40.1%^38^
	Risk of catastrophic expenditure	100% protection	78.8%^37^ 34.3%^38^

Abbreviation: POMR, perioperative mortality rate.

^a^No national POMR system is present to our knowledge. Multiple estimates are provided for postoperative indicators where conflicting information is given by multiple credible sources.

Recognition of local frameworks for health care delivery evaluation is critical, as the local framework is what truly guides national priorities and perspectives. In Kenya, this is the National Health Policy 2014–2030 and the Norms and Standards (N&S) documents, among several others.^5^^–^^7^ These documents define nationally expected and essential health services. Readiness, which is used by the Kenyan Ministry of Health to determine the “capacity of [a] facility to provide specific services,” is a nationally specific statistic for the evaluation of health care delivery through Kenya’s framework for success that can be used to compare across administrative units.^8^ Those expected services depend largely on the level of the health facility.^5^^,^^6^ The reduction of years lived with disability by 25% from 2014 to 2030 is a key policy target, along with a reduction in annual deaths per 1,000 population and increasing life expectancy.^7^ Surgery will play a critical role in achieving these goals, and appropriate investment is essential to realize these targets by 2030. Meeting the expected personnel and service delivery outlined in the N&S would improve access to surgery significantly, but these standards are not always met, as reported in the Kenya Health Readiness Survey.^8^ Contextualizing the current state of surgery in Kenya using both local indicators of success, such as those laid out by the N&S and the Kenya National Health Policy, as well as international indicators provides insight into Kenya’s internal progress and makes comparative studies between Kenya and other countries possible.

Surgery will play a critical role in achieving Kenya's health policy goals, and appropriate investment is essential to realize these targets by 2030.

Health care delivery in Kenya has undergone numerous reforms since gaining independence in 1963.^9^^,^^10^ The most recent major reform is the devolution of service delivery and management to 47 county governments, while the central Ministry of Health coordinates with local authorities on the distribution of funding and supplies and synthesizes guidelines for national health policy.^11^ The Kenya Essential Packages for Health defined a 6-tiered framework for health care delivery, from local community-based health units up to national referral hospitals (Figure).^12^ The level assigned to any given health facility is used to determine the expected service inputs and deliverables. These changes align with the growing recognition within global health that district hospitals, in addition to national hospitals, should play a key role in accessible surgical care delivery.^13^ Benefits of devolution include expanded decision space for human resources for health and improved financial management, but inequalities in access to surgery remain within and between Kenya’s counties.^11^^,^^14^ In this review, we aim to highlight how the LCoGS indicators may be used as a framework to assess progress on developing surgical capacity in a low- and- middle-income country through the lens of Kenya’s surgical system, while also addressing additional factors, such as local policies, that should be included within a national surgical capacity assessment.

**FIGURE.**
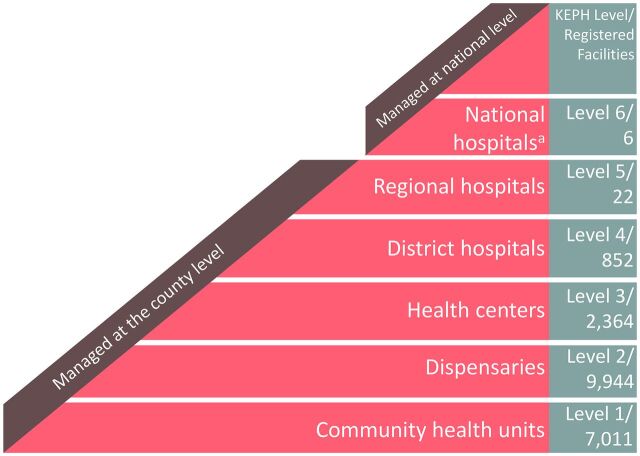
Kenya Health System Framework and Assigned Level and Number of Registered Public and Private Facilities in the Kenyan Facility Master List^15^ Abbreviaton: KEPH, Kenya Essential Package for Health. ^a^Four national hospitals are located in Nairobi.

## METHODS

A narrative review of published literature on surgical capacity in Kenya was conducted by HS to identify relevant articles for LCoGS indicators. Initial PubMed searches on “Kenya*” “surg*” were conducted to establish topic novelty. Articles were initially identified from key papers in global surgery. A snowballing method was used to identify relevant citations and cite articles from key papers. Additionally, we searched PubMed with controlled and free-text searches incorporating terms relating to Kenya, surgery, and each LCoGS indicator. The initial search was supplemented with government and nonprofit reports. Article inclusion was dependent on direct relevance to LCoGS indicators and surgical capacity in Kenya.

## RESULTS AND DISCUSSION

### Preoperative Indicators

Preoperative indicators are key measurements for the quantification of equitable access to surgery across Kenya. The first indicator target—80% coverage of the population with timely access to essential surgery—necessitates several underlying infrastructural and personnel goals: adequate surgical facilities, medicines, and trained SAO personnel for both emergent and elective scenarios. The LCoGS defined the bellwether procedures: laparotomy, cesarean delivery, and open fracture repair, as a proxy measure for whether a facility is capable of a wide variety of surgical presentations. The Table shows established levels of coverage for these. Juran et. al demonstrated that over 90% of Kenyans live within 2 hours of a hospital while assuming that all major hospitals are capable of essential surgery.^16^ However, of surgically equipped hospitals in Kenya, 52% are capable of laparotomy, suggesting that the proportion of hospitals capable of performing all 3 bellwether procedures is 52% at most.^8^

Many questions remain as to the capabilities of those hospitals and to what extent living within 2 hours of a hospital translates to timely care.^17^^,^^18^ For example, the Three Delays framework breaks down barriers to care into 3 categories: delays in care seeking, delays in reaching care, and delays in receiving care, of which the first delay has been found to account for the longest delays in care access.^19^ Challenges in access to maternity services, such as the distance to adequate facilities, remain despite efforts to improve the affordability of these services for impoverished people in rural communities.^20^ As of 2018, 100% of level 5 national referral hospitals and 43% of level 4 hospitals were prepared to offer cesarean deliveries.^8^ Thus, the true proportion of facilities able to perform essential surgical procedures remains unclear, but likely excludes a percentage of Kenyans that, nevertheless, live within 2 hours of a hospital.

The true proportion of facilities able to perform essential surgical procedures remains unclear, but likely excludes a percentage of Kenyans that live within 2 hours of a hospital.

The LCoGS defines the minimum target for the second indicator as 20 SAO personnel per 100,000 population. In 2016, an estimated 2.35 SAO personnel per 100,000 population were working in Kenya.^21^ These estimates are an increase compared to 2014 estimates of SAO personnel per 100,000 people at 0.7 surgeons, 0.3 anesthesiologists, and 0.8 obstetricians, approximately 1.9 SAO personnel per 100,000.^22^ Looking specifically at surgical personnel, a 2016 study estimated the density as 543 surgeons across all specialties, or 1.21 surgeons per 100,000 population, of which 52% were general surgeons and 59% of them were operating in Nairobi, suggesting a concentrated surgical workforce in the capital city.^23^

As such, surgical workforce density captured at a national level does not fully describe the country’s surgical capacity, as the distribution of the surgical workforce may not be proportional to the general population across the country. The 2018 Kenya Harmonized Facilities Assessment estimated that there were on average 1.35 surgeons per district hospital, achieving the N&S goal of 1 surgeon.^8^ However, regional and national hospitals average 2.2 surgeons of the target 8 and 3 surgeons on average of the target 17, respectively.^8^ Nationally, 95% of regional and national hospitals were capable of delivering comprehensive surgical care, with that percentage dropping to 46% of primary hospitals. Additionally, only 30% of secondary and tertiary hospitals and 2% of public primary hospitals offered 24 hours emergency surgery services with a surgeon and anesthetist, suggesting that many hospitals with surgical infrastructure do not have the personnel required to provide emergency care.^8^ While devolution may provide opportunities for more targeted improvements that consider the localized health context, disparities across the country remain.^14^

Several preoperative barriers to effective and equitable SAO care were identified. First, timely access to essential surgical services requires that transportation to an equipped and staffed facility is available. Transit challenges faced by people in need of surgery include road infrastructure, ambulance or motor vehicle access, availability, cost, and adequate communication between providers.^3^ Second, establishing a reliable national blood supply that can provide lifesaving blood products in both urban and rural critical care settings remains a key challenge for surgery in Kenya.^24^ In 2018, a total of 164,275 units of blood were collected compared to a total annual need of 450,000 units.^25^ Finally, surgical tracer items, those supplies recorded by the Kenya Harmonized Facilities Assessment that indicate surgical readiness and include oxygen, scalpels, and sutures, were all present at only 20% of primary public hospitals surveyed.^8^ These additional preoperative barriers remain alongside limited availability of bellwether procedures despite the population’s general proximity to health care facilities in Kenya.

### Perioperative Indicators

Perioperative minimum goals include, first, an annual surgical volume of 5,000 procedures per 100,000 population and, second, the establishment of a system to monitor surgical volume and the POMR. The first indicator demonstrates how the country’s personnel and infrastructure are accessed by the population. The data on surgical volume in Kenya are currently piecemeal or out-of-date (Table). One study estimated that annual surgical volume was 252 per 100,000 population in 1984,^26^ while a 2015 study estimated that Kenya’s surgical volume would be similar to that of other countries with similar economic profiles, concluding that there were 11,110 procedures per 100,000 population per year.^27^ These values are likely under- and overestimates of Kenya’s present-day surgical volume, the true value lying somewhere in between. Indeed, another study estimated that, under 2013 annual operative growth rates, Kenya is estimated to achieve the target of 5,000 procedures per 100,000 population only by 2040–2050.^28^

Current data on Kenya’s surgical volume are likely under- or overestimates of present-day surgical volume.

Specific types of surgery reported by the Ministry of Health include cesarean deliveries and entropion surgeries. The rate of cesarean deliveries, one of the bellwether procedures used to indicate strong surgical capacity, has been increasing in Kenya since 2014, reaching approximately 15.61% of hospital live births at the beginning of 2020.^29^ County-level data suggests that this increase has occurred mostly in urban centers, such as Nairobi, while rural communities still lack access to the surgical resources needed to reach the minimum 5% cesarean delivery rate considered necessary to reduce peripartum mortality.^30^^,^^31^

POMR is a valuable metric for assessing surgical care that allows intra- and international comparative studies of surgical outcomes and safety, so that disparities in care may be identified.^32^ While no study or database documenting POMR at the national level is available for Kenya, several hospital- or cohort-based prospective studies have been conducted. Sileshi et. al found a 7-day POMR of 1.53% across all surgical procedures, with the highest burden being related to general surgery at a 7-day mortality rate of 3.65%.^33^ Newton et. al found pediatric 7-day perioperative mortality was 1.7%, approximately 100 times greater at the 24 Kenyan hospitals included in their study compared to high-income countries.^34^ Nationally, approximately 38% of facilities monitor inpatient mortality secondary to any diagnosis.^8^ An international African study of surgical mortality found a POMR of 1.5% in Kenya, but the authors noted that country-level analysis had low power.^35^ However, without a national surgery registry, extrapolating these available data may not provide an accurate assessment of surgical procedures per capita or the burden of surgically treatable conditions in more remote settings. More robust estimates of Kenya’s POMR require an integrated national system of mortality reporting.

### Postoperative Indicators

Postoperative indicator targets include 100% protection from IE and CHE for patients who receive surgical care. While surgery is a cost-effective way for countries to improve health and productivity, affordable care remains out of reach for many.^27^ In Kenya, an estimated 36.4% of people are at risk of IE due to surgery, defined as out-of-pocket (OOP) payments that push individuals into poverty.^36^ Furthermore, World Bank reports that, in 2020, 21.2% of Kenyans are at risk of CHE, defined as direct OOP payments of more than 10% of the household’s income.^37^ To apply these estimates to the indicator, 63.6% and 78.8% of Kenyans are protected from IE and CHE, respectively (Table). This is an improvement from a 2016 study that estimated the risk of IE and CHE in Kenya was as high as 59.9% and 65.7%, respectively, meaning 40.1% and 34.3% of Kenyans were protected from IE and CHE.^38^ In an analysis of the 2018 Kenya Household Health Expenditure and Utilization Survey, Salari et al. found that approximately 1 million people in Kenya were pushed below the national poverty line by OOP health expenditures, with a greater risk of impoverishment placed on poorer and rural families.^39^ While the authors noted that inpatient expenses generally resulted in fewer cases of CHE, other studies have suggested that this reflects the high cost of inpatient care, which disincentivizes care seeking on the part of poor people most at risk for IE or CHE.^39^^,^^40^ While IE and CHE remain a risk for patients in Kenya, access to surgery will be limited in large part to those who can afford it.

In Kenya, an estimated 36.4% of people are at risk of impoverishing expenditures due to surgery, defined as out-of-pocket payments that push individuals into poverty.

Studies of the true cost of surgical conditions have thus far been piecemeal. For example, Atieno et al. found that cancer care in Kenya placed a significant financial burden on the patient or their families, with surgery costing, on average, KES 128,207 (2021 US$1,196).^41^ In comparison, an estimated 36% of the population is impoverished, having an annual income below KES 3,252 and 5,995 (2021 US$30.33 and US$55.92) depending on rural or urban location.^42^ Poverty in Kenya has been negatively correlated with the usage of hospitals and more advanced health care products.^43^ Because income is linked to education, location, and gender, financial barrier to health care access can translate into disparities on several fronts.

In addition to direct costs, there are many patient expenditures that obscure the true cost of surgery. These additional costs may include costs of transit, OOP payments for diagnostics such as X-rays, cost of inpatient care, medications, and loss of productivity or time at work for the patient and any caretakers.^44^ OOP health expenditures may fall on the patient or the patient’s family members, disincentivizing individuals from seeking care for surgically treatable conditions.^27^ Additionally, the best diagnostic and treatment tools might not be available publicly, forcing patients to seek expensive private care.^45^ While decreasing the personal burden of cost for surgery, it is critical that increased public investment into surgical infrastructure and training is made, and that the tools to measure surgical expenditure are developed and maintained.^46^ As several studies have reported, increasing public investment into surgery is a net economic benefit, despite the misconception that surgery is too expensive for investment to be economically feasible.^47^^,^^48^

### Limitations

This study is limited by the availability of primary research on surgical capacity in Kenya, and extrapolation of estimates from incomplete data sources will likely lead to over- or underestimates of the country’s true SAO situation. It is also possible but unlikely that important data were missed in the process. The LCoGS indicator framework critically does not capture a complete snapshot of national surgical capacity or guarantee that access to surgery is equitable. For example, proximity to a facility capable of the 3 bellwether procedures does capture access to reach the facility but does not guarantee access to receiving the services based on health insurance status or household economic status. In Kenya, much work has gone into developing surgery programs, but more investment is necessary to achieve the targets set by the international community as well as by national authorities. Access to surgery is an essential right—a right that depends on a sufficiently staffed, skilled, and equipped network of facilities that can care for patients regardless of their ability to pay. Kenya has committed itself to policies for universal health coverage with Constitutional guarantees to this right by 2030.^7^ However, given the predicted timing of achievement of the LCoGS in 2040–2050^28^ and the impact of COVID-19 on households and the health system,^49^^,^^50^ the challenging policy environment threatens to further delay SAO targets.

## CONCLUSIONS

Here, we have reviewed surgical capacity in Kenya through the surgery indicator framework developed by LCoGS.^3^ These 6 indicators, when assessed altogether, provide insight into a country’s SAO capacity and infrastructure. Local and national health priorities must be considered to align national and international development goals or sustainable development of surgical capacity will not be possible. Nationally relevant data, for example, Kenya’s Harmonized Facilities Assessment surgical readiness measurement,^8^ provides additional insight into the state of surgical care in Kenya that dovetails with international indicators. Therefore, Kenyan national health policy documents were reviewed for measures of surgery readiness.

While national estimates for each indicator provide some insight into Kenya’s current surgical capacity, they may also reduce the complexity inherent in a devolved health care system to statistics that do not capture disparities felt across the country.^14^ Development of county-level infrastructure to monitor surgical volume and perioperative outcomes would better inform governments tasked with allocating funds for health system development through targeted and strategic funding schemes. Additional metrics, such as transfusion availability, ambulance availability, referral statistics, and availability of on-call SAO personnel could be beneficial for determining the true readiness and capacity for surgery. The adoption of a National Surgical, Obstetrics, and Anesthesia Plan for Kenya could clarify the targets for SAO care, provide infrastructure for the measurement of those targets, and outline strategies for the expansion of surgical services. A Kenyan National Surgical, Obstetrics, and Anesthesia Plan could integrate ideas implemented in Ethiopia and Tanzania with the local needs and context.^51^^,^^52^

While national estimates for each indicator provide some insight into Kenya’s current surgical capacity, they may also reduce the complexity inherent in a devolved health care system to statistics that do not capture disparities felt across the country.

Surgery remains a key area for development in global health with potential leverage points for improvements in quality of life that have not been taken advantage of historically. Reduction of the burden of surgically treatable conditions is beneficial not only for the patient whose quality of life is improved but also for the productivity and economic potential of the nation.^53^ Studies into the risk of IE and CHE have highlighted the need for free or affordable surgery through government policies that recognize the direct costs of receiving care.^3^^,^^38^ This is reflected by the incorporation of direct OOP expenditure into SAO indicators, but the incorporation of indirect medical costs into surgery system indicators is also essential. A policy that considers only direct OOP medical costs will exclude patients who must pay for transportation, food, and housing, among other costs to access necessary surgery. As Kenya looks toward universal health coverage, studies have found that insurance may be positively, rather than inversely, correlated to IC and CHE, further strengthening the need for health financing systems that consider all potential costs and barriers to care seeking.^39^^,^^54^^–^^56^ Beyond Kenya, the LCoGS indicators as a tool for assessing surgical capacity can be easily implemented in other settings. Findings from Kenya possibly reflect the situation in similar lower-middle-income countries. Further, the LCoGS framework allows for systematic comparisons to be made between countries. A useful application would be a tool for stakeholders to recognize regions that are leaders in developing surgical capacity and those that require additional investment and attention.

## Notes

**Peer Reviewed**

**First published online:** February 18, 2022.
